# Electrospinning of Flexible Poly(vinyl alcohol)/MXene Nanofiber-Based Humidity Sensor Self-Powered by Monolayer Molybdenum Diselenide Piezoelectric Nanogenerator

**DOI:** 10.1007/s40820-020-00580-5

**Published:** 2021-01-16

**Authors:** Dongyue Wang, Dongzhi Zhang, Peng Li, Zhimin Yang, Qian Mi, Liandong Yu

**Affiliations:** 1 College of Control Science and Engineering, China University of Petroleum (East China), Qingdao, 266580 People’s Republic of China; 2 State Key Laboratory of Precision Measurement Technology and Instruments, Department of Precision Instruments, Tsinghua University, Beijing, 100084 People’s Republic of China

**Keywords:** Self-powered sensing, Monolayer molybdenum diselenide, Piezoelectric nanogenerator, Humidity sensor, Flexible electronics

## Abstract

Two-dimensional material has been widely investigated for potential applications in sensor and flexible electronics. In this work, a self-powered flexible humidity sensing device based on poly(vinyl alcohol)/Ti_3_C_2_T_x_ (PVA/MXene) nanofibers film and monolayer molybdenum diselenide (MoSe_2_) piezoelectric nanogenerator (PENG) was reported for the first time. The monolayer MoSe_2_-based PENG was fabricated by atmospheric pressure chemical vapor deposition techniques, which can generate a peak output of 35 mV and a power density of 42 mW m^−2^. The flexible PENG integrated on polyethylene terephthalate (PET) substrate can harvest energy generated by different parts of human body and exhibit great application prospects in wearable devices. The electrospinned PVA/MXene nanofiber-based humidity sensor with flexible PET substrate under the driven of monolayer MoSe_2_ PENG, shows high response of ∼40, fast response/recovery time of 0.9/6.3 s, low hysteresis of 1.8% and excellent repeatability. The self-powered flexible humidity sensor yields the capability of detecting human skin moisture and ambient humidity. This work provides a pathway to explore the high-performance humidity sensor integrated with PENG for the self-powered flexible electronic devices.

**Supplementary Information:**

The online version contains supplementary material available at(10.1007/s40820-020-00580-5)

## Highlights

- A flexible piezoelectric nanogenerator (PENG) based on 2D single-layer MoSe_2_ flake on polyethylene terephthalate was fabricated.
- A high-performance flexible poly(vinyl alcohol)/MXene (PVA/MXene)-based humidity sensor was fabricated by electrospinning.
- The PVA/MXene composite-based humidity sensor was self-powered by MoSe_2_ PENG and exhibited excellent properties.

**Supplementary Information:**

The online version contains supplementary material available at(10.1007/s40820-020-00580-5)

## Graphical abstract

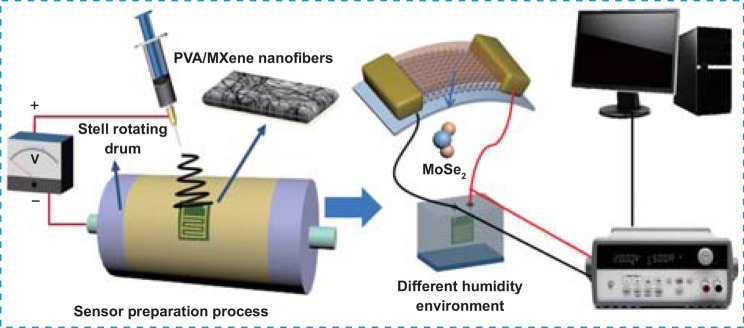

## Introduction

Humidity sensor has become increasingly indispensable in many areas such as industrial manufacture, medical health, and air quality monitoring, especially in the Internet of Things and flexible electronics [1]. At present, many kinds of signal detection technique have been developed and applied to humidity detection, including capacitance [2], quartz crystal microbalance [3], bulk acoustic wave (BAW) [4], and surface acoustic wave (SAW) [5]. However, these detection techniques all require complex detection equipment and power source, which increases application cost and energy consumption. The traditional method is using the batteries to drive humidity sensors to work, which limits the wide range of application for sensors in Internet of Things. Usually, a small amount of power can make most types of sensors operate [6]. In view of the large amount of available clean energy existing in the surrounding environment or human body, we can harvest these energies to build sustaining self-powered sensing system [7–9]. Therefore, it is expected to develop a simple and low-cost humidity sensing system without external power supply through self-powered technology [10, 11].

The latest technologies for collecting energy mainly include piezoelectricity [12, 13], triboelectricity [14–17], pyroelectricity [18], photoelectricity [19], and electromagnetism [20]. Among these technologies, nanogenerator based on triboelectricity and piezoelectricity is considered to have excellent application prospects due to its high durability and mechanical stability, especially in the field of self-powered sensors [21]. A piezoelectric nanogenerator (PENG) prepared from zinc oxide nanowires was first reported by Wang et al. in 2006, which gained great concern because of its excellent piezoelectric performance [22]. Liu et al. designed a self-powered multifunctional monitoring system using hybrid integrated triboelectric and piezoelectric microsensors, which can effectively monitor the relative humidity (RH) level and carbon dioxide concentration [23]. Zhang et al. reported a novel self-recovering triboelectric nanogenerator (TENG) as an active multifunctional sensor. The device has a wide humidity detection range (20%–100% RH) and rapid response/recovery time (18/80 ms) [24]. Xia et al. designed a conductive copper tape-based TENG combined with LiCl for humidity detection. The TENG has a power density of 240.1 μW cm^−2^, and the RH can be represented by the brightness of the LEDs driven by the TENG [25]. Tai et al. developed an air-driven triboelectric nanogenerator based on Ce-doped ZnO-PANI, which was used to detect the NH_3_ concentration, flow rate, and frequency of exhaled gas [26]. Many new nanomaterials with excellent piezoelectric properties were continuously studied. Monolayer boron nitride (BN), MoS_2_, MoSe_2_, WTe_2_, WSe_2_, and MoTe_2_ have been theoretically predicted to exhibit piezoelectric property [27]. And it has been experimentally confirmed that single-layer MoS_2_ showed piezoelectric effect and was applied to PENG [28, 29].

In recent years, two-dimensional (2D) nanomaterials such as graphene, metal oxides, transition metal dichalcogenides (TMDs), metal organic frameworks (MOFs), black phosphorus have attracted tremendous interests due to their excellent physical, chemical, and electrical properties. Especially, 2D nanomaterials have been employed in constructing high-performance sensors and flexible electronic devices [30, 31]. In 2011, MXene was first synthesized by Yury et al. [32]. As a new 2D nanomaterial, MXene exhibits high specific surface area, high conductivity, and excellent flexibility, which is considered to have great application prospects in humidity and wearable sensors. Lu et al. found alkalized MXene exhibited much better sensing properties toward NH_3_ and humidity, compared with untreated MXene [33]. Due to excellent metal conductivity and hydrophilicity, MXene not only exhibits more excellent gas and humidity sensing properties, but also a very promising material for building flexible sensors to provide excellent mechanical stimulus sensing performance. Wang et al. reported a piezoresistive flexible sensor prepared by MXene/natural microcapsule, which has a fast response time (14 ms), satisfactory repeatability, and stability [34].

In this work, a self-powered flexible humidity sensor based on electrospinned poly (vinyl alcohol)/Ti_3_C_2_T_x_ (PVA/MXene) nanofibers film and monolayer molybdenum diselenide (MoSe_2_) piezoelectric nanogenerator was reported for the first time. The monolayer MoSe_2_ PENG was fabricated on a flexible polyethylene terephthalate (PET) substrate. The PVA/MXene nanofibers film was prepared on the interdigital electrodes (IDEs) as the humidity-sensitive material through electrospinning technology. The prepared self-powered piezoelectric humidity sensor (PEHS) was driven by the monolayer MoSe_2_ PENG via converting mechanical energy to electric energy. The self-powered PVA/MXene nanofibers film humidity sensor has a large response, fast response/recovery time, low hysteresis, and excellent repeatability. Furthermore, the humidity sensing mechanism of PVA/MXene sensor was explored.

## Experiment

### Materials

Hydrochloric acid (HCl, analytical purity) and lithium fluoride (LiF, 99%) were purchased from Sinopharm Chemical Reagent. Titanium aluminum carbide powders (Ti_3_AlC_2_) and poly (vinyl alcohol) (PVA) were from Shanghai Macklin Biochemical Technology.

### Materials Synthesis

Synthesis of MXene: LiF (1 g) was dispersed in a polypropylene plastic bottle with 6 M HCl solution (20 mL) and then was stirred for 5 min to ensure a fully dissolution. One gram of Ti_3_AlC_2_ was added slowly to the mixed solution to avoid violent reaction of the solution, followed by placed at 35 °C for 24 h. The product after reaction was washed with deionized water several times until the pH of supernatant is greater than or equal to 6. And then, the product was treated via centrifugation at 3500 rpm for 5 min, and the dark green supernatant collected as delaminated Ti_3_C_2_T_x_.

Preparation of PVA/Ti_3_C_2_T_x_ mixed suspensions: Electrospinning is a typical fiber manufacturing process that can produce fibrous nanofilms with larger specific surface area [35, 36]. One gram of PVA was placed in 9 g of deionized water to obtain 10% (w/w) PVA solution. The solution was stirred for 3 h at 90 °C. Then, the PVA/MXene solution was prepared by adding 0.1 g of Ti_3_C_2_T_x_ into PVA solution and magnetic stirring for about 0.5 h.

### Fabrication of the PENG and PEHS

**Fig. 1:**
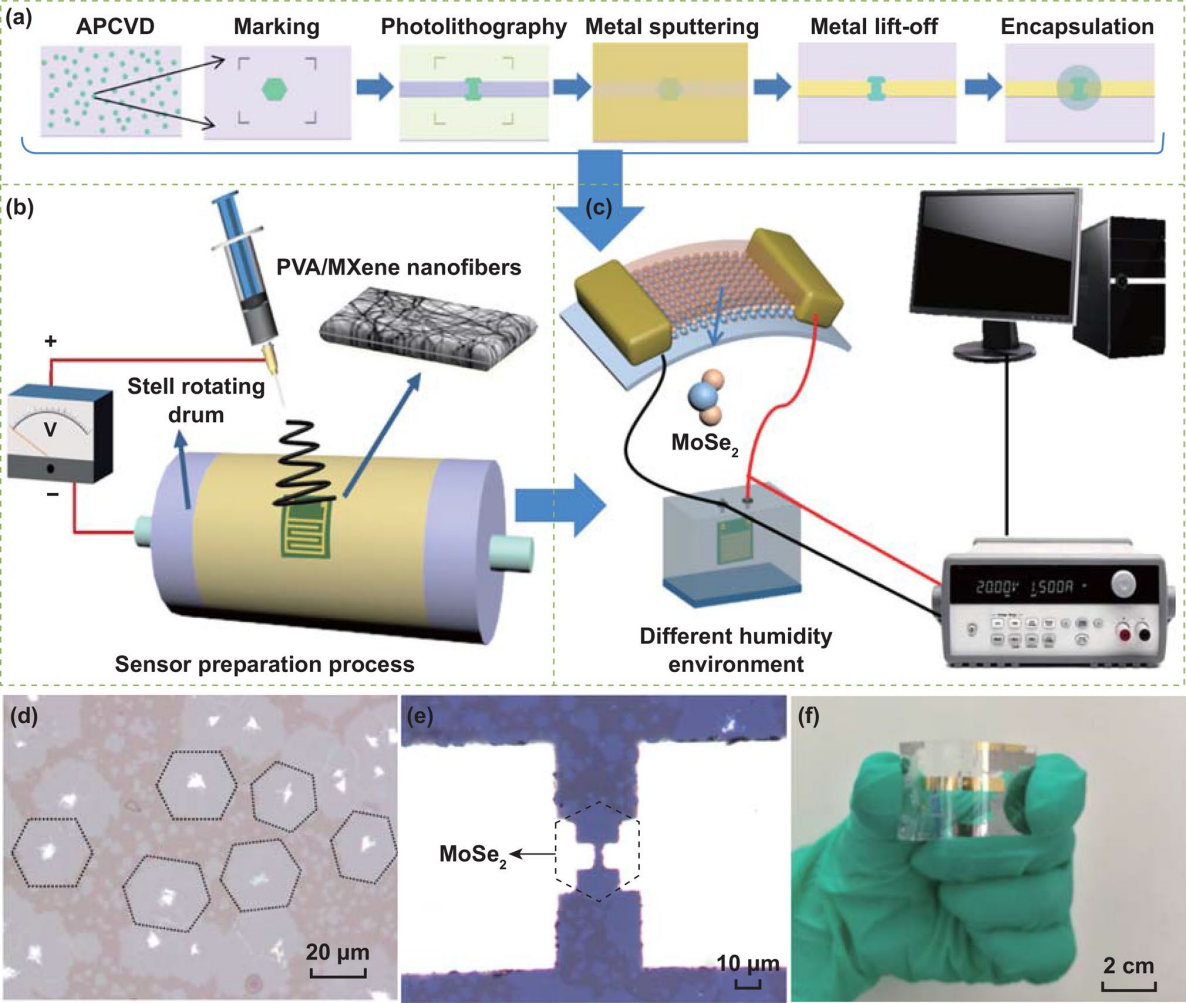
Schematic diagram for the fabrication of **a** MoS⁠e_2_-flake-based PENG and **b** PVA/MXene humidity sensor. **c** Schematic of the experimental platform for humidity sensing measurement. **d** Monolayer MoSe_2_ prepared by APCVD. **e** Optical microscope image of a MoSe_2_ piezoelectric device with two electrodes. **f** Photograph of the flexible PENG device

Monolayer MoSe_2_ flake was employed for the fabrication of the PENG. Figure 1a shows the schematic diagram for the fabrication of PENG. The electrodes were prepared by photolithography, metal deposition (10 nm Cr/100 nm Au), and lift-off process. As shown in Fig. 1d, e, the monolayer MoSe_2_ prepared by atmospheric pressure chemical vapor deposition (APCVD) method was transferred to flexible PET substrate and exhibits irregular hexagon, followed by packaging with polydimethylsiloxane (PDMS) film. The white dots in the middle are the centers of the crystal nucleus. The monolayer MoSe_2_ on PET is sealed by PDMS film, which is isolated from the external environment to avoid the influence of environment humidity. Figure 1f shows the photograph of the flexible PENG device. The monolayer MoSe_2_ flakes connected with Au electrodes along with armchair are performed. And the MoSe_2_ atomic orientation was identified by optical second harmonic generation (SHG) as in Fig. S1.

As shown in Fig. 1b, the humidity sensor with PVA/MXene nanofibers film was prepared on interdigital electrodes (IDEs) with epoxy substrate using electrospinning technology. The dimension of IDEs is 8 × 8 mm^2^, and the thickness is 0.3 mm. Electrospinning is widely used for preparing continuous nanofibers from viscoelastic fluids through electrostatic repulsion force between surface charges. Electrospinning nanofibers have the advantages of small porosity, high porosity, and large specific surface area, and are promising building blocks for the fabrication of sensors. The applied voltage between positive and negative poles was 18 kV, and the needle-to-collector distance was 15 cm. The flow rate is 0.3 mL h^−1^, and the duration is 0.5 h. Schematic of the experimental platform for humidity sensing measurement is shown in Fig. 1c. PENG is driven by a tensile testing machine (MIT-1021). The humidity sensor was driven by PENG via converting mechanical energy into electrical energy. The humidity sensing properties were systematically investigated under a humidity range of 11–97% RH at 25 °C. The output voltage of PENG and PEHS was measured by a digital multimeter (Keysight 34470A).

### Characterization Instrument

The structure and surface morphology of the PVA/MXene nanofibers were characterized using scanning electron microscopy (SEM, Hitachi S–4800, Japan) and transmission electron microscope (TEM, Jeoljem-2100, Japan). The X-ray diffractometer (XRD, Rigaku Miniflex 600) with CuKα radiation (*λ* = 0.15418 nm) was used to investigate its crystal structure. The MoSe_2_ atomic layer was characterized by confocal Raman microscopy (Horiba HR–800) with a laser wavelength of 514 nm. Fourier transform infrared spectroscopy (FTIR) spectra were recorded using a PerkinElmer Spectrum Two FTIR spectrometer.

## Results and Discussion

### Fundamental Measurement of PENG

**Fig. 2:**
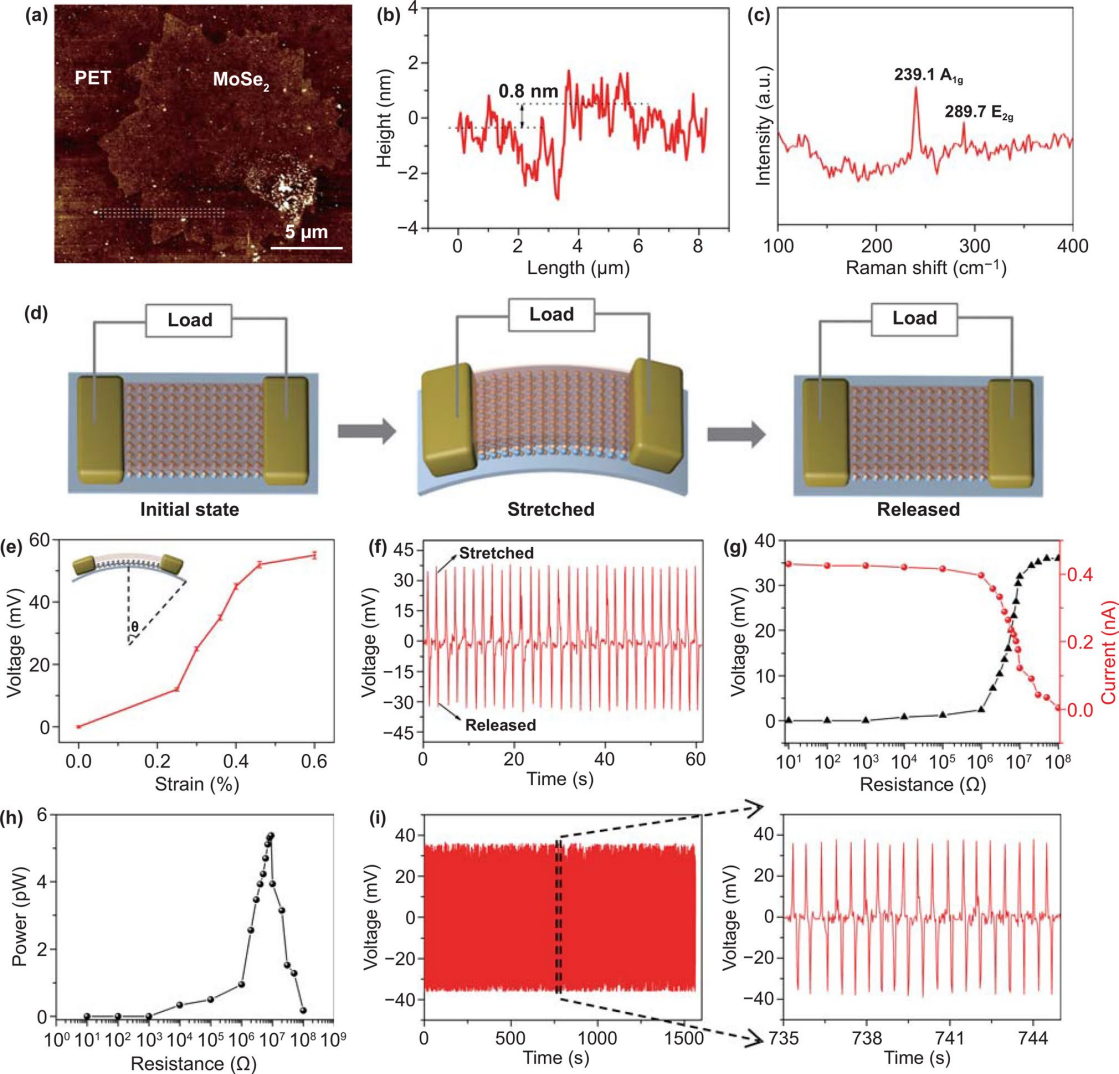
**a** AFM topographic image of the single-layer MoSe_2_ flake. **b** Relative heights along the white lines in Fig. 2a. **c** Raman spectrum of the monolayer MoSe_2_ flake. **d** Operation scheme of the monolayer MoSe_2_ piezoelectric device. **e** Open-circuit voltages of the device as a function of strain. **f** Real-time output voltage under 0.36% strain at a frequency of 0.5 Hz. **g** Dependence of output voltage and current from a monolayer MoSe_2_ device under 0.36% strain as a function of external loading resistance. **h** Power versus the loading resistance. **i** Cyclic test showing the stability of monolayer MoSe_2_ device for prolonged period

The thickness of monolayer MoSe_2_ was identified by AFM. Figure 2a shows the AFM topographic image of the hexagon-like-shaped monolayer MoSe_2_ flake, and Fig. 2b exhibits the corresponding height profile, which is about 0.8 nm for the monolayer MoSe_2_ structure [37]. Figure 2c shows the characterization result using Raman spectrum. The prepared MoSe_2_ has two major characteristic peaks at 239.7 and 289.7 cm^−1^, which are corresponding to the out-of-plane A_1g_ and in-plane E_2g_ modes, respectively [38]. Figure 2d shows the operation scheme of the MoSe_2_ piezoelectric device. No induced charge was generated at both ends of the MoSe_2_ flake in the initial state. When the PENG was stretched, a monolayer of MoSe_2_ would generate charges with opposite polarities at its edges. When the PENG was released, reverse electron flow resulted in a negative voltage peak. Periodic stretching and releasing can cause PENG produces alternating positive and negative voltage output signals (Video S1). The piezoelectric output of a MoSe_2_ PENG is related to the magnitude of the applied strain. Figure 2e shows the relationship between open-circuit voltages and the strain of the device. The strain was defined as Eq. (1) [29]:

1 $$\varepsilon = \frac{h}{2R}$$

where *h* is the thickness of the flexible substrate, *R* is the radius of curvature when the PENG was stretched. It can be found that the output voltage rises as the degree of the applied strain increases. The output voltage can reach 55 mV at strain of 0.6%, which is much higher than monolayer MoS_2_ PENG [28]. Figure 2f shows the real-time output voltage (35 mV) under 0.36% strain at a frequency of 0.5 Hz. As shown in Fig. S2, PENG is bent at different frequencies. The bending frequency has little effect on the voltage output of PENG. In subsequent experiments, we apply strain of 0.36% to avoid MoSe_2_ slippage.

Figure 2g shows the output voltage and current from the PENG at 0.36% strain under different loading resistances. The output current decreases slowly in low resistance range (1 Ω–100 MΩ) and then declines rapidly with the increasing load, and the voltage changes with the opposite trend. Figure 2h shows the output power under different loading resistances. The maximal output power of PENG is up to 5.37 × 10^−9^ mW at a loading resistance of 9.2 MΩ and a power density of about 42 mW m^−2^, which is higher than other kinds of PENGs (Table 1) [28, 39–42]. As shown in Fig. 2i, the cyclic test shows the good stability of PENG for prolonged period and indicates that the energy conversion is stable.

**Table 1:** Performance of the MoSe_2_ PENG in this work compared with the previous work

Piezoelectric material	Open-circuit voltage (mV)	Short-circuit current (nA)	Power density (mW m^-2^)	Refs.
ZnO NRs	2.8	8500	0.08	[39]
PVDF-TrFE	17	~	~	[40]
SnS_2_ nanosheet	33	0.18	1.14	[41]
KNN piezo-resin/CFRP	2	~	0.16	[42]
MoS_2_ nanosheet	15	0.02	2	[28]
MoSe_2_ nanosheet	35	0.4	42	This paper

### Energy Harvesting of Human Activities

**Fig. 3:**
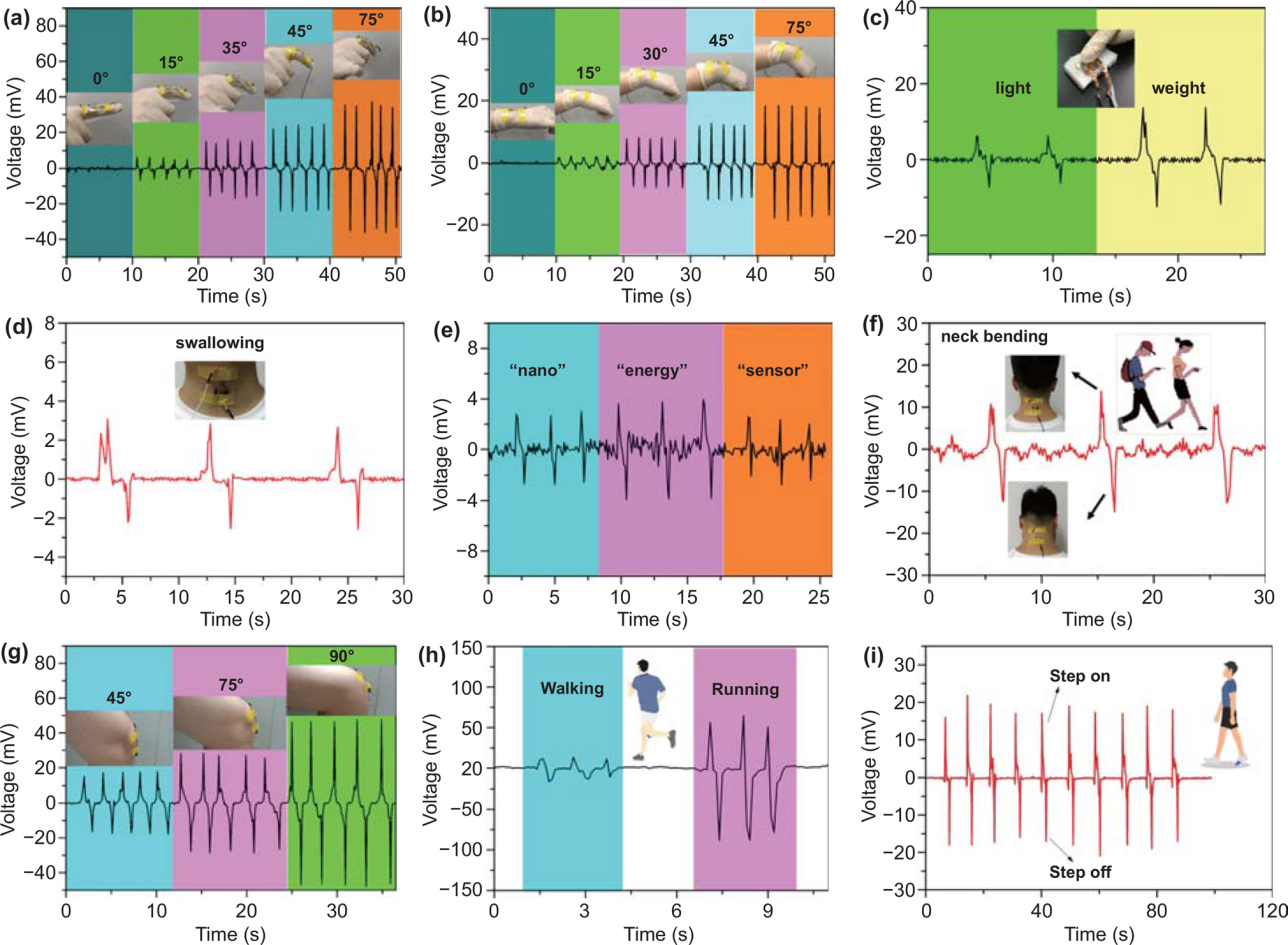
Energy harvesting and application of MoSe_2_ device on various parts of the human body **a** Index finger joint. **B** Wrist. **c** Finger press. **d** Throat. **e** Output voltage change of flexible PENG in terms of various sound stimuli, such as “nano,” “energy,” and “sensor.” **f** Neck bending. **g** Knee bending. **h, i** Human motion detection

We further explored the energy harvesting of MoSe_2_ device on various parts of the human body. Figure 3a illustrates the energy harvesting at the knuckles (Video S2). The device is attached to the finger joint and bends with the finger. We can observe that the PENG generates different output voltages for wrist bending motions at five different angles of 0°, 15°, 30°, 45°, and 75°. When the bending degree increases, the PENG exhibits an enhanced output voltage. The similar experiment was performed with the device attached to the wrist in Fig. 3b. The bending of the wrist produces a smaller output voltage compared to finger under the same angle. This could be because the knuckles cause a greater degree of bending of flexible PET. The device was placed on a sponge and applied different pressures with fingertip. Figure 3c shows the output voltage under different pressures. The pressure can be identified by detecting the output voltage. Figure 3d shows that the device can identify swallowing action of the throat. And based on this, we detect the relative voltage change of MoSe_2_ flexible device by making different kinds of sounds, such as “nano,” “energy,” and “sensor” (Fig. 3e). As shown in Fig. 3f, we also detected the output voltage based on neck bending when the device was attached to the nape. Considering that neck diseases of an increasing number of teenagers due to looking down and playing with mobile phones, this application will have great prospects in the future. Human body is mainly driven by legs to walk and run. Figure 3g–i shows the energy harvesting when the device was attached to the knee and sole of the foot; the device can stably collect the energy generated by the legs and identify different movements like walking and running.

### Characterization of PEHS

**Fig. 4:**
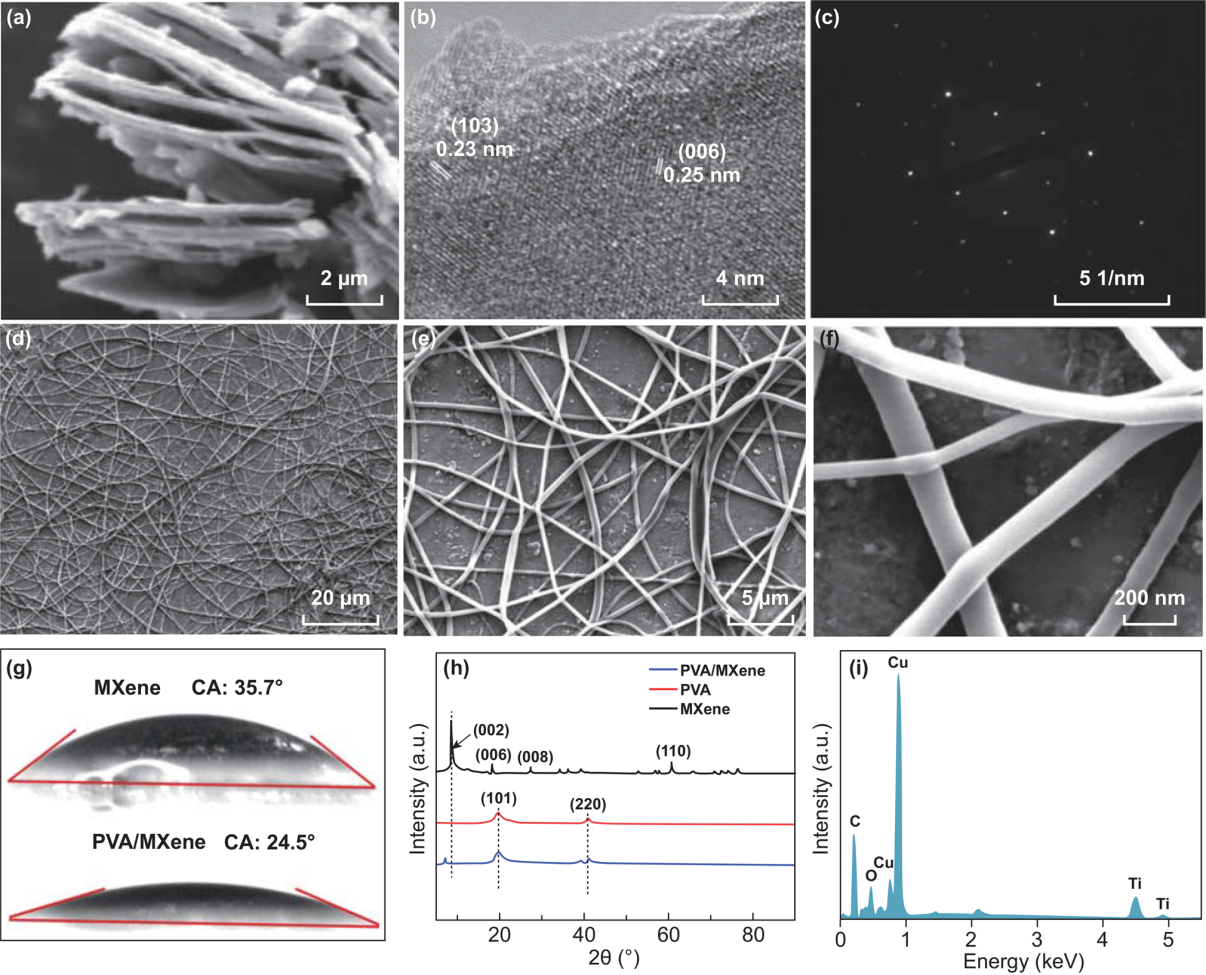
**a** SEM of MXene multilayer structure. **b** TEM images of few-layer MXene nanoflakes. **c** Corresponding selected-area electron diffraction (SAED) pattern of the hexagonal arrangement of atoms. **d, e** SEM images of PVA/MXene nanofibers. **f** Contact angle measurement of MXene and PVA/MXene. **g** XRD, **h** EDS, and **i** FTIR pattern of MXene, PVA, and PVA/MXene composite

Figure 4a shows the SEM of MXene multilayer structure; the small particles between layers may be the broken MXene or TiO_2_ [43]. Figure 4b, c shows the TEM images of few-layer MXene nanoflakes and the corresponding selected-area electron diffraction (SAED) pattern of the hexagonal arrangement of atoms, respectively. The SAED exhibits the hexagonal arrangement of atoms. As shown in Fig. 4d–f, the SEM images show that the PVA/MXene nanofibers are successfully prepared. The average fiber diameter of PVA/MXene nanofibers is 170 nm. As shown in Fig. 2g, the water contact angles of MXene and PVA/MXene were 35.7° and 24.5°, respectively. The PVA/MXene has the minimal contact angle and exhibits excellent hydrophilicity. The XRD characterization results of PVA, MXene, PVA/MXene are illustrated in Fig. 4h. The XRD pattern of MXene shows four prominent peaks at 2θ = 8.4°, 18.1°, 26.8°, and 60.6°, which are assigned to the (002), (006), (008), and (110) planes [32]. The XRD pattern of pure PVA shows two character peaks at 2θ = 19.4° and 41.2°, which are attributed to the (101) and (220) planes [44]. And it can be found that PVA did not destroy the crystal structure of MXene from the XRD pattern of PVA/MXene. The (002) peak for PVA/MXene is downshifted from 2θ = 8.4° to 2θ = 7.2° as compared to that of MXene; this change is considered to be due to the increase in the distance between the MXene nanosheets causing by the deposition of PVA molecules [36].

Figure 4i shows energy-dispersive spectrometer (EDS) images of PVA/MXene nanofibers film. The Ti and O elements are derived from MXene and PVA, respectively, and the C element comes from both MXene and PVA. The Cu is derived from copper foil substrate. The FTIR characterization results of PVA, MXene, PVA/MXene are illustrated in Fig. S4. The wide peak for all materials at about 3440 cm^−1^ is O–H stretching vibration peak. The other typical peaks of PVA are at 2930 cm^−1^ for C–H stretch, 1720 cm^−1^ for C=O stretch, 1631 cm^−1^ for –C–C– stretch, 1095 cm^−1^ for C–O stretch, respectively [45]. The other typical peaks of MXene are observed at 1710 and 550 cm^−1^, which are assigned to the stretching vibration of C–O and O–H, respectively [46]. The FTIR characterization result of PVA/MXene shows no obvious broad peak or shifts after the addition of MXene [47].

### Humidity Sensing Properties of PEHS

**Fig. 5:**
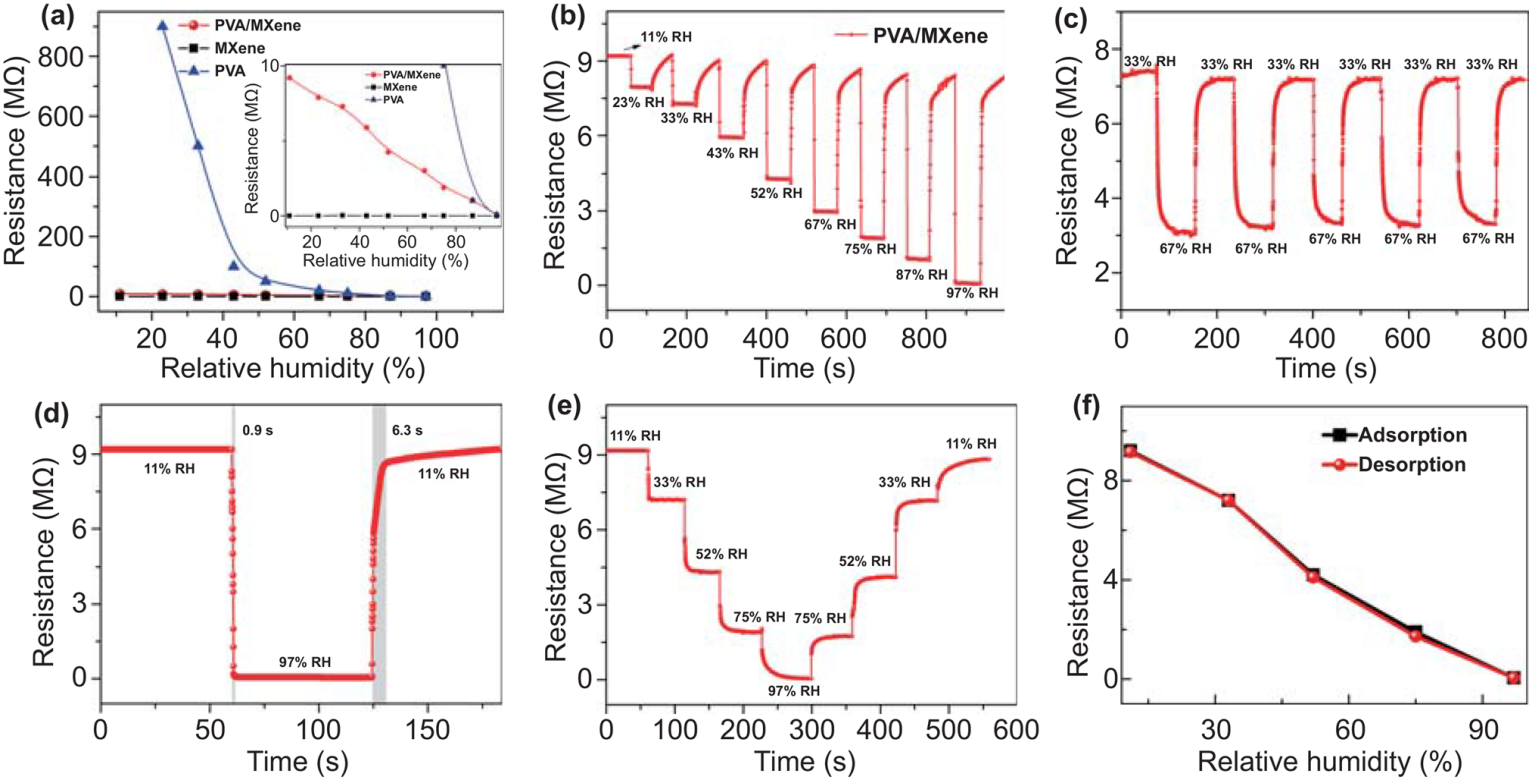
**a** Resistance of the MXene, PVA, and PVA/MXene film sensor exposed to various relative humidities. **b** Dynamic resistance changes of PVA/MXene film sensor exposed to various relative humidities. **c** Repeatability of PVA/MXene film sensor. **d** Time-dependent resistance response and recovery curves of the PVA/MXene sensor between 11 and 97% RH. **e** Resistance of sensor with increasing and decreasing humidity. **f** Humidity hysteresis curves of the PVA/MXene nanofibers film sensor

The resistances of MXene, PVA, PVA/MXene sensors at different humidity levels are shown in Fig. 5a. Different humidity levels are provided by corresponding saturated salt solutions [48]. The resistance variation range (0.08–9.2 MΩ) of PVA/MXene sensor is aligned with the rising *U-R* (voltage-resistance) region of PENG (the U–R curve in Fig. 2g). The PVA sensor exhibited a high resistance state (≥ 80 MΩ) over a wide humidity range (11–52% RH), and the MXene sensor showed a small resistance change (2.1–3.3 kΩ), which are not conducive to the combination of the sensor and PENG as a self-powered humidity sensor. Therefore, PVA/MXene sensor driven by the prepared PENG is easier to obtain the corresponding relationship between output voltage and humidity levels. Figure 5b shows the dynamic resistance change of the PVA/MXene sensor exposed to various RHs. The response/recovery time is defined as the time when the sensor achieved 90% of total resistance variation. Figure 5c, d shows that the sensor has good repeatability and fast response/recovery time (0.9/6.3 s), respectively. As shown in Fig. 5e, f, we investigated the hysteresis characteristic of PVA/MXene sensor versus RH. The sensor hysteresis is defined as H = (*R*_A_–*R*_D_)/*S* (%RH). *R*_A_ and *R*_D_ are the sensor resistance in the adsorption and desorption process of water molecules, and S is the sensor sensitivity. We investigated the hysteresis characteristic of PVA/MXene sensor versus RH, which shows the PVA/MXene humidity sensor has low hysteresis of 1.8%. The sensor has excellent humidity sensing performance, which is attributed to the humidity sensitivity of composite material. MXene has strong hydrophilicity and high electrical conductivity. The resistance of MXene film increases with the increase in humidity level, which may be the result of the increase in layer spacing caused by water molecules embedded in the MXene layers [49, 50]. The conductivity change of PVA/MXene can be caused by the adsorption of water molecules under humidity environment. Both PVA and MXene contain a large number of hydroxyl groups (–OH), and the proton can transition between two adjacent hydroxyl groups. In addition, protons can help electron transfer between water molecules. Under low humidity level, proton-assisted electron tunneling is the main reaction process. Under high humidity level, water molecules are firmly bound to hydroxyl groups. Hydrogen ions formed by PVA adsorbed water molecules hop between water molecules. With the increase in humidity, the concentration of hydrogen ions increases, resulting in the decrease in sensor resistance. In addition, MXene can be used as the charge transmission and conduction layer of composite materials due to its excellent metallic conductivity, which is conducive to accelerating the adsorption/desorption process of water molecules. Therefore, the PVA/MXene sensor achieved a fast response/recovery behavior [51, 52].

**Fig. 6:**
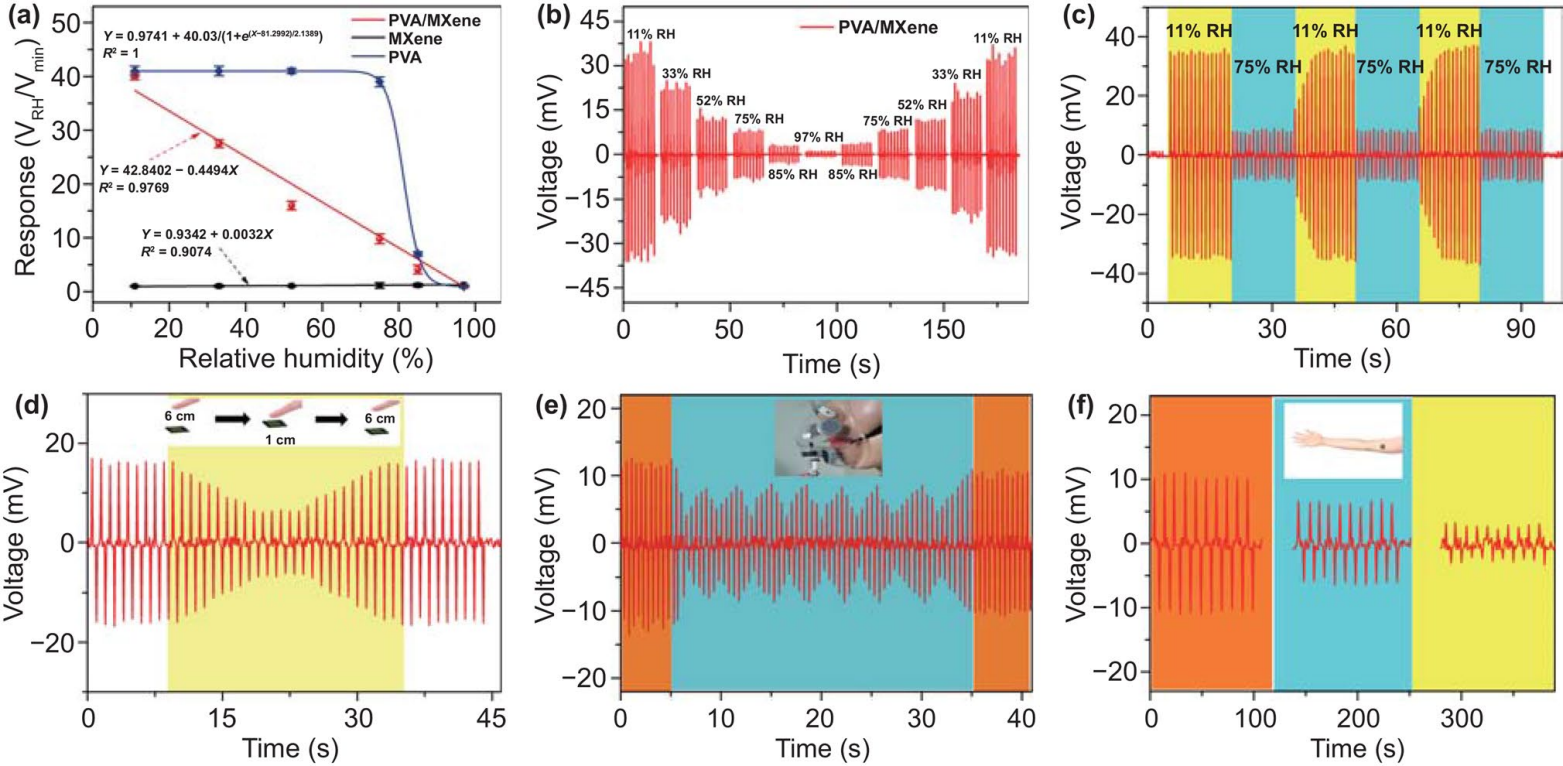
**a** Response fitting curves of self-powered MXene, PVA, and PVA/MXene film sensors toward different humidities. **b** Output voltage for PVA/MXene-based PEHS upon exposure to different humidities. **c** Repeatability of self-powered PVA/MXene sensor. **d** Output voltage when the finger slowly approaches the sensor. **e** Flexible sensor is used to detect human breathing rate. **f** Test result of detecting the humidity of arm skin surface after different exercise times

Figure 6a shows the response–humidity fitting curves of MXene, PVA, and PVA/MXene sensors at 11–97% RH. The actual voltage value is shown in Fig. S3a. The minimum value of output voltage is the reference value of the sensor response. The response of humidity sensor was defined as: *S*=*V*_RH_/*V*_min_, where the *V*_RH_ is the output voltage of sensor at the target humidity and the *V*_min_ is the minimum output voltage. The PVA sensor has the same voltage output in the humidity range of 11–52% because of its large resistance, and the voltage output of MXene sensor is close to 0 because of its small resistance. The experimental results are consistent with the previous analysis results; neither PVA nor MXene sensor is suitable to combine with PENG to detect humidity. The prepared self-powered PVA/MXene sensor holds high humidity response of ∼40. The corresponding equation is Y = 42.8402−0.4494X, and the regression coefficient (R^2^) is 0.9769. Figure 6b shows the output voltage for PVA/MXene nanofibers film sensor driven by PENG when sensor exposed to wide humidity range (11–97% RH). It can be seen that the output voltage exhibits highest value at 11% RH and has obvious decrease with increasing humidity. The different humidity levels can be distinguished by particular output voltage. The self-powered PVA/MXene nanofiber film sensor has excellent repeatability as in Fig. 6c. There is no obvious change in output voltage by comparing test result. And we also measured the output voltage when the finger slowly approaches the sensor as in Fig. 6d. Finger approaches the sensor at a constant speed (0.5 cm s^−1^) at a distance from the sensor (6 cm) and then leaves at the same speed. Table 2 summarizes the humidity sensing performance of the presented PEHS in comparison with previous works [24, 25, 53, 54]; the comparison highlights the PEHS has much higher response in a wide RH range. Figure S3b shows the long-term stability of PVA/MXene nanofibers film sensor driven by PENG over a period of 30 days. It can be found that the sensor has no noticeable voltage drift and exhibits excellent stability. Figure 6e shows the output voltage when the flexible sensor is used to detect human breathing rate. Figure 6f shows the test result of detecting the humidity of arm skin surface after different exercise times when the sensor is attached to the arm. The output voltages all occur regular changes. Thus, the flexible humidity sensor driven by PENG exhibits excellent performance on detecting human skin surface moisture and has great application prospects in wearable devices.

**Table 2:** Performance of the presented sensor in this work compared with the previous work

Sensor materials	Meas. range	Response	Response/recovery time	Refs.
RGO/PVP	7–97.3% RH	7	2.8/3.5 s (90%)	[53]
LiCl	40–80% RH	12	~	[25]
Ga/ZnO	45–80% RH	4	5 s (90%)	[54]
PTFE/Al	20–100% RH	28	18/80 ms (~)	[24]
PVA/MXene	11-97% RH	40	0.9/6.3 s (90%)	This paper

## Conclusions

In this work, a self-powered humidity sensing device based on monolayer MoSe_2_ PENG has been proposed for the detection of humidity. The piezoelectric properties of monolayer layer MoSe_2_ were reported at first time. A high peak output of 35 mV can be obtained when the PENG was under 0.36% strain at a frequency of 0.5 Hz. And the flexible PENG can harvest energy and generate different output voltages by attaching to different parts of human body. The self-powered sensor was prepared by PVA/MXene composite nanofibers film and driven by the monolayer MoSe_2_ PENG to detect humidity by converting mechanical energy to electric energy, which has a larger response (40) and 40-fold higher than pure MXene. And the humidity sensor also shows fast response/recovery time of 0.9/6.3 s, low hysteresis of 1.8%, and stable repeatability. Moreover, the PVA/MXene nanofibers film was also used to prepare flexible humidity sensor on a PET flexible substrate and exhibited excellent performance on detecting human skin surface moisture.

## Supplementary Information

Supplementary file 1 (DOC 1440 kb)

Supplementary file 2 (MP4 11087 kb)

Supplementary file 3 (MP4 13225 kb)
