# Supplementary material for: Electrospinning of Flexible Poly(vinyl alcohol)/MXene Nanofiber-Based Humidity Sensor Self-Powered by Monolayer Molybdenum Diselenide Piezoelectric Nanogenerator
Source: Nanomicro Lett. 2021 Jan 16;13:57. doi: 10.1007/s40820-020-00580-5 (PMC8187675; doi:10.1007/s40820-020-00580-5)
Supplement: Supplementary file 1 — (DOC 1440 kb) [file 40820_2020_580_MOESM1_ESM.doc]

**Supporting Information**

Electrospinning of Flexible Poly(vinyl alcohol)/MXene Nanofiber-Based Humidity Sensor Self-Powered by Monolayer Molybdenum Diselenide Piezoelectric Nanogenerator

**Dongyue Wang^a^, Dongzhi Zhang^a^,*, Peng Li^b^,*, Zhimin Yang^a^, Qian Mi^a^, Liandong Yu^a^,***

^a^ College of Control Science and Engineering, China University of Petroleum (East China), Qingdao 266580, China

^b^ State Key Laboratory of Precision Measurement Technology and Instruments, Department of Precision Instruments, Tsinghua University, Beijing 100084, China

*Corresponding author: Dongzhi Zhang, Peng Li, Liandong Yu

E-mail address: [dzzhang@upc.edu.cn](mailto:dzzhang@upc.edu.cn), [pengli@mail.tsinghua.edu.cn](mailto:pengli@mail.tsinghua.edu.cn), liandongyu@upc.edu.cn

Tel: +86-532-86982928

Fax: +86-532-86983326

**
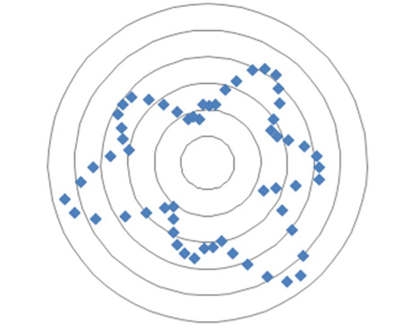
**

**Fig. S1** The SHG result of monolayer MoSe_2_.


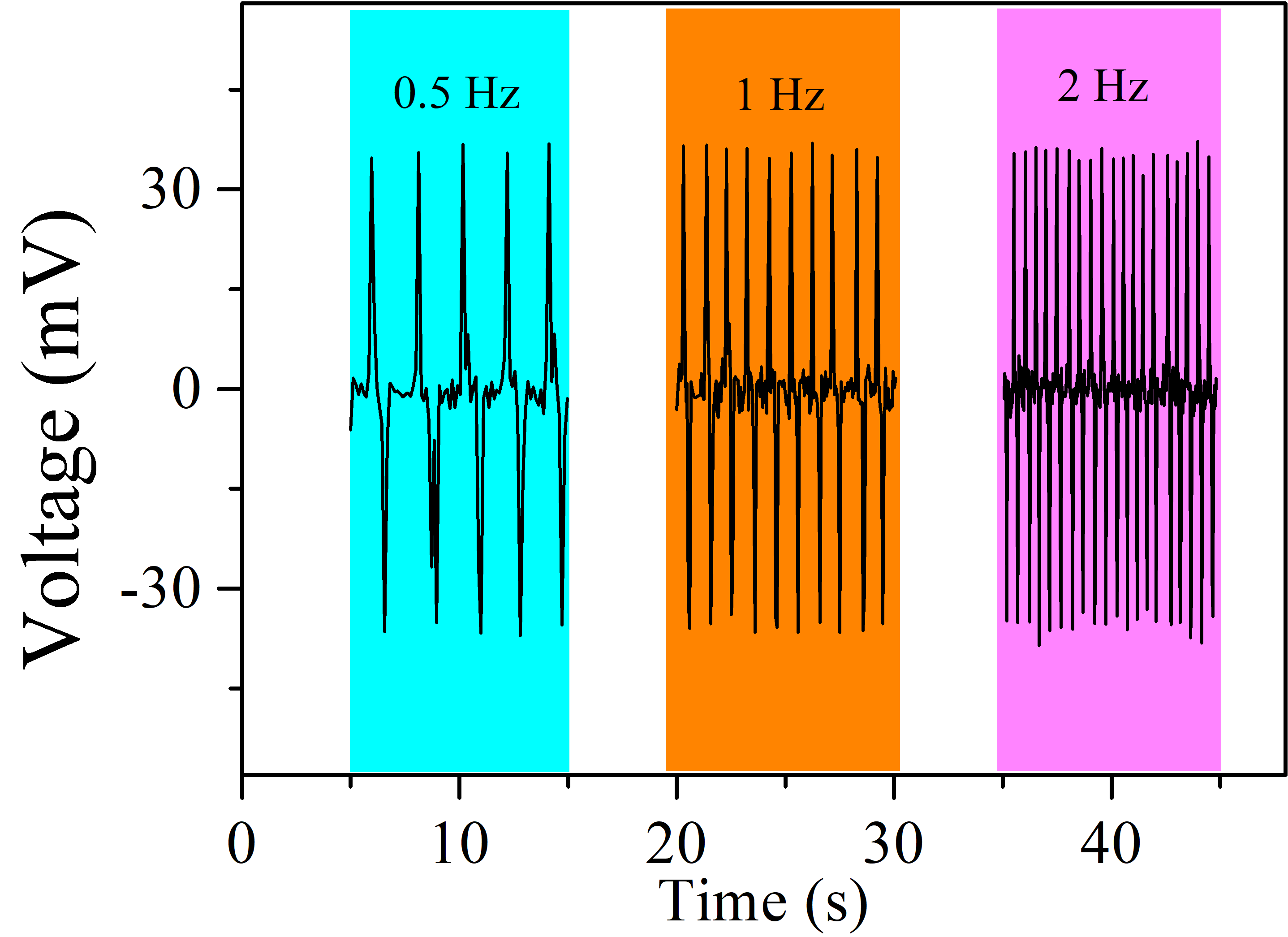


**Fig. S2** The output voltage of PENG under different bending frequencies.


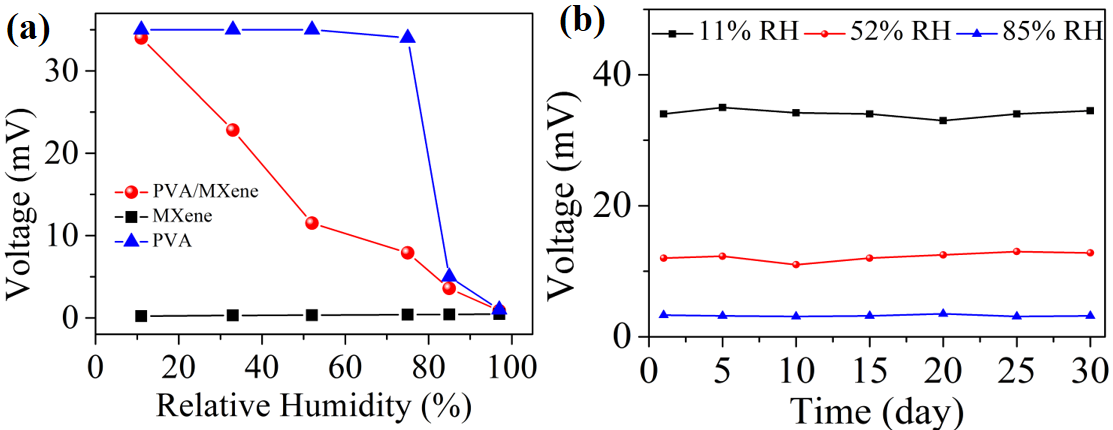


**Fig. S3** **a** The actual voltage value of self-powered MXene, PVA and PVA/MXene sensor. **b** The long-term stability of PVA/MXene nanofibers film sensor driven by PENG.


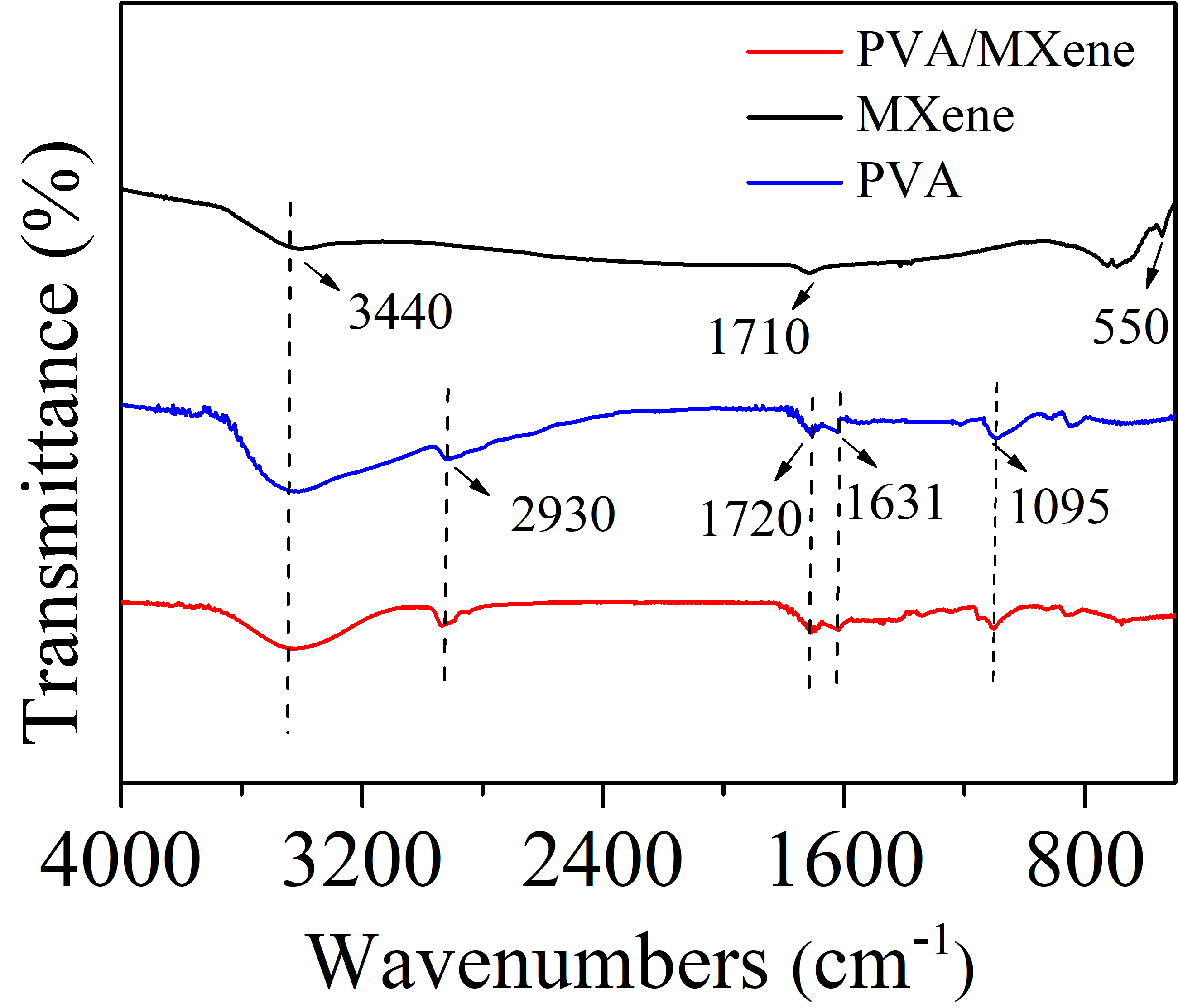


**Fig. S4** The FTIR characterization results of PVA, MXene, PVA/MXene.
